# Emergency department use in the postpartum period: a retrospective cohort study

**DOI:** 10.21203/rs.3.rs-4014132/v1

**Published:** 2024-03-12

**Authors:** Elijah Reische, Mark Santillan, Victoria Cunningham, Kelsey Blocklinger, Stephen Hunter, Elissa Faro, Heather Davis, Boyd Knosp, Donna Santillan

**Affiliations:** University of Iowa Health Care; University of Iowa Health Care; University of Iowa Health Care; University of Iowa Health Care; University of Iowa Health Care; University of Iowa Health Care; University of Iowa Health Care; University of Iowa Health Care; University of Iowa Health Care

**Keywords:** emergency department, postpartum, race, C-section, pregnancy

## Abstract

**Background:**

Visits to the emergency room (ED) by women in the postpartum period may reflect gaps in postpartum care and disparities in access to obstetric and primary care services. This study aimed to characterize the patients who visited the ED in the first year after delivery, their reasons for coming to the ED, and the care they received.

**Methods:**

The electronic health record was reviewed for all patients who delivered at University of Iowa Health Care between 2009 and 2023 and visited the ED within 365 days after delivery. Data drawn directly from the EHR included patient demographics and medical history, pregnancy and delivery information, and newborn characteristics. The charts were then reviewed manually for information regarding ED visits including time from delivery, chief complaint, diagnosis, and disposition.

**Results:**

555 pregnancies had ED visits within one year of delivery, with a total 814 ED visits across the study sample. 46.7% of ED visits occurred in the first 30 days following delivery, and 35% of ED visits for obstetric complaints occurred in the first 2 weeks after delivery. Black patients visited the ED more often (mean=1.84 visits, SD=1.30) than white (mean=1.34, SD=0.92, p<0.001) or Hispanic patients (mean=1.35, SD=0.67, p = 0.004). The most common categories of chief complaint were obstetric (34.6%) and gastrointestinal (18.8%), while the most common categories of diagnosis were obstetric (31.8%) and immune/infectious (28.1%).

**Conclusions:**

Visits to the ED are common in the year following delivery. Almost half of these visits occur in the first 30 days after birth. The plurality of postpartum ED visits are due to obstetric complaints, especially in the first few weeks. Black women are more likely to use the ED during this period, potentially due to disparities in healthcare access. These findings suggest that some of these ED visits may be preventable, and that there is room for improvement in post-delivery follow-up, communication between patients and the obstetrics team, and access to outpatient obstetric care.

## BACKGROUND

In 2022, 3.7 million births took place in the United States (1). Many women develop complications in the weeks and months following delivery, and many of these complications result in visits to the emergency department (ED). Within the first 6 weeks after birth, about 5% of women will present to the ED (2).

The reasons for these visits to the ED are highly varied. They include pregnancy-related problems, medical illness unrelated to pregnancy, and psychiatric emergencies. Previous research has found that some of the most common pregnancy-related reasons for ED visits include vaginal bleeding, C-section incision complications, and breast concerns (3, 4, 5). Common reasons for ED visits unrelated to pregnancy include trauma, headache, and non-obstetric infection (2). Psychiatric emergencies may be related to pregnancy (e.g. postpartum depression or psychosis) or unrelated.

Not all pregnant women are equally likely to present to the ED in the postpartum period. There are a number of risk factors for postpartum ED utilization, many of which are associated with reduced access to healthcare. These include race, public insurance, mental illness, and rural location. Other risk factors are related to medical history, including cesarean section (C-section), preexisting comorbidities, and delivery complications (4, 6, 7, 8, 9).

Understanding the causes and risk factors for postpartum ED visits is important for a number of reasons. First, visits to the ED are costly, both for patients and for health systems. In 2019, the mean cost for a visit to the ED was $615. Costs for ED visits have also been increasing steadily over time (10). This represents increased financial demands on insurance companies, patients, government aid, and hospitals. Additionally, many patients present to the ED for complaints that would be better addressed in an outpatient clinic setting or even at home. Unnecessary visits to the ED place an additional burden on a system that is already over capacity, often resulting in long wait times and poor patient experience. Some ED visits made in the postpartum period may not be strictly necessary and could be preventable.

This study aimed to characterize ED visits in the postpartum period and the patients making those visits at a large midwestern academic medical center in order to better understand why they occur and if some of them may be prevented.

## METHODS

### Study approval:

Institutional Review Board approval was obtained at the University of Iowa (IRB# 201902830).

### Participation Criteria:

Patients were included who delivered between 2009–2023 and had at least one visit to the University of Iowa Health Care (UIHC) ED within one year of delivery. Exclusion criteria included patients under 18 years old and/or prisoners.

### Data acquisition:

The electronic health record was queried for deliveries that occurred at UIHC between 2009 and 2023 and were associated with visits to the UIHC ED within 365 days after delivery. Charts were reviewed and data were extracted both automatically and manually. Data points collected included information about the birthing parents, including demographic information, payer type, obstetric history, pregnancy complications, and delivery interventions. Rural-urban commuting area (RUCA) codes were used to classify the population density of the area in which each patient lived (11). Data were also collected on the newborns, including birth measurements, Apgar scores, and hospital disposition following birth. Data points collected regarding the ED visits included days from delivery, chief complaint, consults to obstetrics and gynecology or family medicine, diagnosis, and disposition. Chief complaints were classified into organ system categories, and obstetric (OB) chief complaints were further specified. Diagnoses were also classified into organ system categories, and specific diagnoses were collected as well. Visits to outside EDs were not included unless the patient was transferred to UIHC. Data were stored using REDCap (Research Electronic Data Capture) (12).

### Analysis:

Primary outcomes of interest included number of ED visits in the first year postpartum, days from birth to the first ED visit, chief complaint at presentation to the ED, consults placed in the ED, ED diagnosis, and disposition. To identify risk factors for repeat postpartum ED visits, number of ED visits was compared between patients by race, RUCA code, parity, and delivery type using non-parametric Kruskal-Wallis tests. Time from birth to first ED visit was compared between patients by race, RUCA code, and delivery type using non-parametric Kruskal-Wallis tests. Chief complaints were compared by delivery type and time to first ED visit using chi-square tests. Consults were compared by delivery type using chi-square tests. Disposition from the ED was compared by ED chief complaints as well as various pregnancy complications using chi-square tests. Statistical analysis was performed using R.

## RESULTS

**Figure 1:**
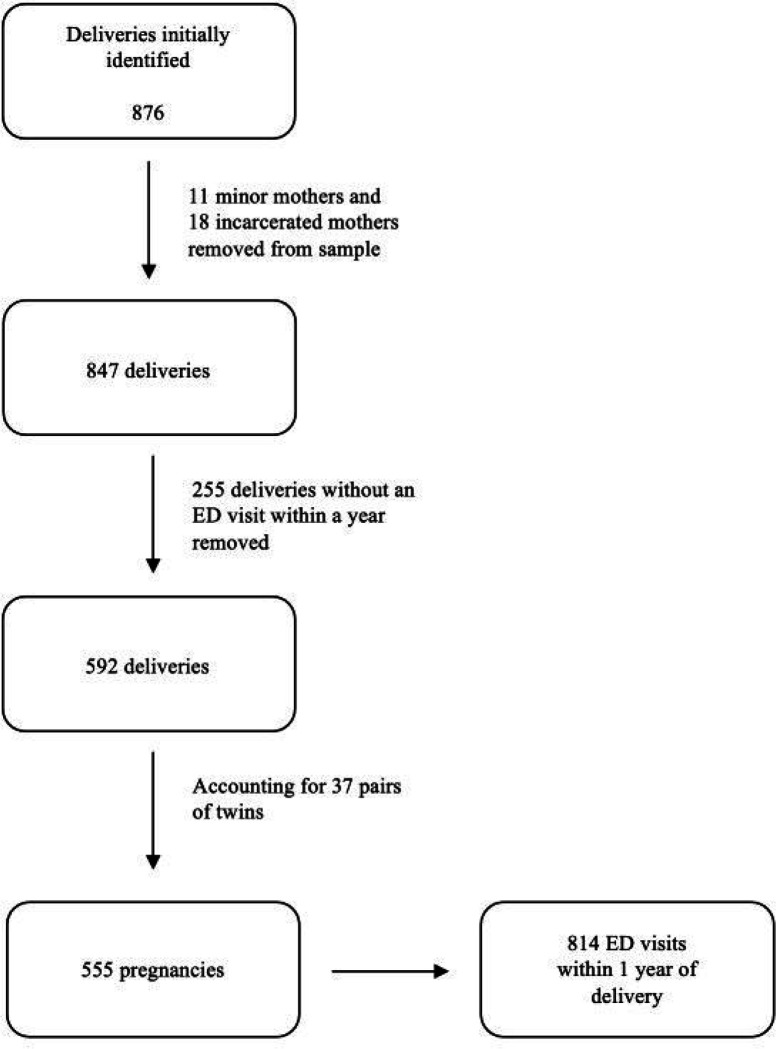
Selection of the study sample.

The original sample included deliveries of 876 newborns. Deliveries by 11 minor patients and 18 incarcerated patients were removed from the sample. 255 of these deliveries were found not to have had an ED visit in the following year, bringing the final sample size to 592 deliveries. Accounting for multiple gestations, the total number of pregnancies in the study was 555. A total of 814 ED visits occurred in the first year following these pregnancies. (Fig. 1)

Population characteristics of the women are presented in Table 1. The mothers had an average age of 28.9 +/− 5.8 years at delivery. 61% were white, 23% were Black, 9% were Hispanic, 4% were Asian, 2% were multiracial, and 1% were American Indian/Alaska Native. Mean gravidity was 3.04+/− 1.88 and mean parity was 1.30 +/− 1.40. The most common RUCA code was 1, corresponding to “metropolitan area core: primary flow within an urbanized area,” with 327 patients living in these areas.

Pregnancy, delivery, and newborn characteristics are shown in Table 2. Of the 555 pregnancies included in the study, 521 were singletons and 34 were twins. Mean gestational age at delivery was 36.8 wks +/− 4.3 wks. The majority of the patients underwent assisted rupture of membranes (52%), with 35% rupturing membranes spontaneously. Mean duration of ruptured membranes was 22.7 hours. The majority of the deliveries were spontaneous vaginal deliveries (56%), followed by C-sections (39%) and assisted vaginal deliveries (5%). Patients remained in the hospital for a mean of 4.6 days. 10.5% of patients had a gestational diabetes diagnosis, and 11.7% had a pre-eclampsia diagnosis.

The mean birth weight of the newborns was 2875g +/− 981g. 54% of newborns had 1 minute Apgar scores of 8 or 9, and 83% had 5-minute scores of 8 or 9. 51% were admitted to the newborn nursery following delivery, while 21% were admitted to the neonatal intensive care unit.

**Figure 2:**
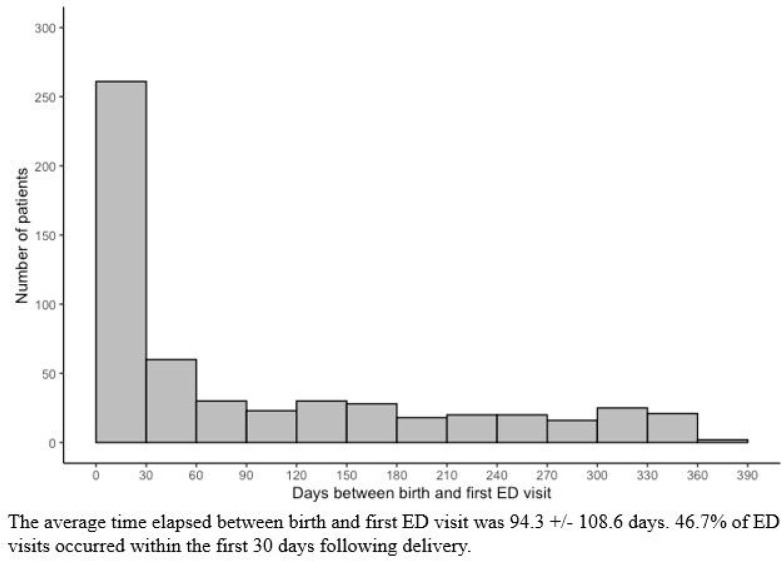
Days from birth to first ED visit.

The average number of ED visits in the first year postpartum was 1.5 +/− 1.0. The average time from delivery to the first ED visit was 94.3 +/− 108.6 days. 46.7% of ED visits occurred within the first 30 days following delivery (Fig. 2). Most common ED chief complaints, including most common OB-related chief complaints, can be seen in Table 3. OB was the most common category of chief complaint, accounting for 34.6% of ED visits. Of patients with OB chief complaints, the most common specific complaints were lower abdominal pain (34.4%) and vaginal bleeding (31.2%). Gastrointestinal was the next most common category at 18.8% of patients. 24.8% of patients had complaints in multiple categories. The Obstetrics and Gynecology service was consulted in person for 15.9% of patients, and by telephone for 5.4% of patients. Family medicine was consulted in person for 0.61% of patients, and by telephone for 0.25% of patients. Most common ED diagnoses are listed in Table 4. The most common category of diagnosis was, again, obstetric (31.8%) followed by immune/infectious (28.1%). 32.3% of patients had diagnoses in multiple categories. Most common specific diagnoses were urinary tract infection (UTI) (4.2%), mastitis (3.1%), and endometritis (3.1%).

The average time elapsed between birth and first ED visit was 94.3 +/− 108.6 days. 46.7% of ED visits occurred within the first 30 days following delivery.

**Figure 3:**
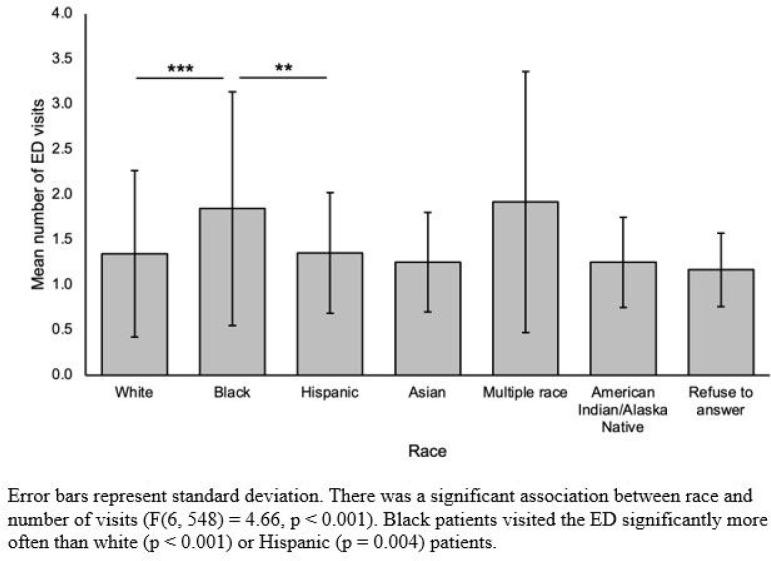
Number of ED visits within 1 year of delivery by race.

Number of ED visits was significantly associated with race (F(6, 548) = 4.66, p < 0.001, Fig. 3), with Black patients visiting the ED significantly more than white (p < 0.001) or Hispanic (p = 0.004) patients. There was no significant association between number of ED visits and RUCA, insurance payer type, parity, or delivery type.

Error bars represent standard deviation. There was a significant association between race and number of visits (F(6, 548) = 4.66, p < 0.001). Black patients visited the ED significantly more often than white (p < 0.001) or Hispanic (p = 0.004) patients.

There was a significant association between mode of delivery and time from birth to first ED visit (F(3, 549) = 7.96, p < 0.001). Time to first ED visit was also significantly associated with diagnosis category (X^2^ = 110.5, p = 0.001), with 35% of visits for obstetric diagnoses occurring in the first 2 weeks following delivery. There was no association between time to first ED visit and race or RUCA code.

There was a significant association between delivery type and having an obstetric chief complaint (X^2^ = 11.47, p = 0.02), with patients who had assisted vaginal deliveries, dilation and evacuation procedures, and spontaneous abortions more likely to have obstetric chief complaints than patients who had spontaneous vaginal deliveries or C-sections. Furthermore, there was a significant association between delivery type and specific obstetric chief complaint (X^2^ = 194.86, df = 56, p < 0.001, Table 4).

Disposition from the ED had significant associations with pregnancy complications. 45.5% of patients with pre-eclampsia were admitted to the hospital compared to 21.7% of patients without preeclampsia (X^2^ = 34.6, df = 10, p = 0.0001). 37.5% of patients with eclampsia were admitted to the hospital, compared to 23.5% of patients without eclampsia (X^2^ = 58.0, df = 10, p < 0.0001). 22.2% of patients with gestational diabetes were admitted to the hospital compared to 34.9% without gestational diabetes (X^2^ = 24.2, df = 10, p = 0.007) during pregnancy. Furthermore, there was a significant association between disposition and chief complaint (X^2^ = 460.5, df = 140, p < 0.0001), with increased likelihood of hospital admission for patients with psychiatric (53%), respiratory (32%), and neurologic (24%) chief complaints compared to other categories. Of patients who presented with obstetric chief complaints, 72% were discharged home.

83 patients (15%) were pregnant at the time of their ED visit. The most common chief complaint category for these patients was obstetric (63%). The most common obstetric chief complaints among pregnant patients were multiple complaints (25% of total pregnant patients), vaginal bleeding (22%), and lower abdominal pain (17%). Women who were pregnant at the time of presentation were significantly younger (27.3 +/− 4.5 years) than those who were not (29.4 +/− 5.8 years) (W = 12830, p = 0.005).

7 patients (1.3%) presented to the ED immediately after delivering precipitously at home or on the way to the hospital. Women living in an area with a RUCA code of 8 (small town, high commuting) were significantly more likely to present after delivery (X^2^ = 36.7, df = 8, p < 0.0001). However, only 1 patient who presented immediately after delivery lived in one of these areas.

## DISCUSSION

Visits to the ED in the postpartum period are common, with 5% of women presenting in the first 6 weeks following delivery alone (2). In our study, which examined the first year following delivery at the only academic medical center in a rural state, nearly half of the ED visits occurred in the first month. Obstetric chief complaints were the most common, with a large proportion of visits for these complaints occurring in the first 2 weeks. These early visits to the ED for obstetric complaints may represent a target for prevention of postpartum ED visits as they are most likely to be preventable by increased patient education by the delivering team or by the providers providing postpartum or discharge care. Patients who underwent C-sections presented to the ED sooner on average than those who delivered vaginally, suggesting an increased rate of complications or potentially a gap in care for these patients.

Most patients who presented to the ED in the first year postpartum went only once or twice, but Black women made more ED visits on average than white or Hispanic women. There are a number of potential explanations for this disparity. Black women are at an increased risk for postpartum complications (13), and thus may be more likely to present to the ED for those symptoms. Furthermore, due to systemic racism, many Black patients lack access to primary care due to location, insurance status, and discrimination by healthcare providers (14, 15). As a result, Black patients in general visit the ED more often and are more likely to use the ED for primary care due to lack of access (16). Postpartum outreach programs targeted to Black women, as well as more generalized efforts to improve primary care access for Black patients, may reduce the frequency of ED visits among this marginalized population.

The most common category of both chief complaint and eventual diagnosis for postpartum women visiting the ED was obstetric, with a large proportion of these visits occurring in the weeks immediately following birth. Nearly ¾ of women presenting to the ED for obstetric complaints were discharged to home. The most common specific diagnoses made in the ED were mastitis, endometritis, and UTI. These are all fairly common problems following delivery (17, 18, 19) and can often be managed in an outpatient setting with oral antibiotics (or supportive care, in the case of mastitis) in uncomplicated cases (18, 20, 21). Some of these visits may have been preventable by improving patient education regarding what to expect during the postpartum period, who to contact for specific issues, and what severity of symptoms necessitates an ED visit. Women whose deliveries were classed as dilation and evacuation procedures, spontaneous abortions, or assisted vaginal deliveries were more likely to present to the ED for obstetric complaints. These patients may be at higher risk for postpartum complications and necessitate a clinic or telephone follow-up within 1–2 weeks.

About 15% of patients were pregnant again at the time of their ED visit, meaning they had an interpregnancy interval shorter than 1 year. Interpregnancy intervals shorter than 18 months are associated with an increased risk of adverse outcomes, and many of the patients in this study presented with vaginal bleeding and lower abdominal pain. 19 (22.9%) of these patients were found to be having a spontaneous or threatened abortion. As this subset of patients was significantly younger than non-pregnant patients, this may indicate a need to improve education for younger patients on how long to wait between pregnancies, as well as access to postpartum contraception for young patients.

This study aimed to characterize the population of women visiting the emergency department in the postpartum period. One of the study’s strengths was its comprehensiveness, as it included every single postpartum ED visit at the UIHC within the time period studied. Additionally, UIHC is a major tertiary care center, a safety-net hospital, and the only academic hospital in the state, and therefore draws patients from a variety of locations and backgrounds. This project also extended the period of study up to 1 year postpartum. Consequently, these results provide a broader view of postpartum ED utilization than previously published studies, as most earlier studies have only examined up to 6 weeks postpartum. Due to privacy limitations, we were unable to collect data on ED visits at other institutions. Therefore, our results are likely an underestimation of true ED utilization among the study sample as patients may have gone to a local ED for care. The use of local ED care also could bias our results to artificially lower apparent ED usage by rural patients who live a greater distance from UIHC. Future work may expand upon this study by analyzing treatment given at the ED. Understanding what treatments beyond hospitalization were provided could assist in clarifying whether patients are visiting the ED for problems that could be addressed in an outpatient setting.

## CONCLUSIONS

Among patients presenting to the ED in the first year postpartum, nearly half present in the first month following delivery, and the greatest proportion present for obstetric complaints. Black women visited the ED more frequently during this period than Hispanic or white women. Many patients were diagnosed with common postpartum problems including mastitis and UTI. Patients who were pregnant again at the time of their ED visit were younger than those who were not pregnant. These findings represent potential target populations for improvements of postpartum care.

## Figures and Tables

**Table 1:** Baseline characteristics of mothers involved in the study.

	Mean (numeric data) or count (categorical data)	Standard deviation (numeric data) or percentage (categorical data)
Age (years)	29	5.7
Race		
White	337	61%
Black	128	23%
Hispanic	48	9%
Asian	20	4%
Multiple race	12	2%
AI/AN	4	1%
Refuse to answer	6	1%
Ethnicity		
Not Hispanic or Latino	488	87.9%
Hispanic or Latino	60	10.8%
Other	5	0.9%
Unknown	2	0.4%
RUCA code		
1 (Metropolitan area core)	326	58.7%
2 (Metropolitan area high commuting)	47	8.5%
3 (Metropolitan area low commuting)	1	0.2%
4 (Micropolitan area core)	64	11.5%
5 (Micropolitan high commuting)	5	0.9%
6 (Micropolitan low commuting)	0	0.0%
7 (Small town core)	57	10.3%
8 (Small town high commuting)	5	0.9%
9 (Small town low commuting)	1	0.2%
10 (Rural areas)	49	8.8%
Gravidity	3.04	1.88
Parity	1.3	1.4
Term births	1.13	1.32
Preterm births	0.16	0.43
Abortions	0.67	1.07
Therapeutic abortions	0.09	0.37
Spontaneous abortions	0.47	0.89
Ectopic pregnancies	0.03	0.25
Positive drug screen	16	2.9%

AI/AN = American Indian/Alaska Native, RUCA= Rural Urban Commuting Area.

**Table 2:** Characteristics of pregnancies, deliveries, and newborns.

	Mean (numeric data) or count (categorical data)	Standard deviation (numeric data) or percentage (categorical data)
Newborn count		
Singleton	521	93.9%
Twin	34	6.1%
Gestational age (wks)	36.81	4.34
Gestational diabetes	58	10.5%
Pre-eclampsia	65	11.7%
Rupture of membranes type		
Assisted	291	52.4%
Spontaneous	195	35.1%
Premature	30	5.4%
Intact	30	5.4%
Unknown	9	1.6%
Delivery type		
Spontaneous vaginal	310	55.9%
C-section	214	38.6%
Assisted vaginal	27	4.9%
Dilation and evacuation	3	0.5%
Spontaneous abortion	1	0.2%
Birthweight (g)	2875.1	980.8
Apgar score (1 minute)		
0	21	3.5%
1	14	2.4%
2	11	1.9%
3	22	3.7%
4	24	4.1%
5	28	4.7%
6	53	9.0%
7	90	15.2%
8	165	27.9%
9	148	25.0%
Unknown	16	2.7%
Apgar score (5 minutes)		
0	19	3.2%
1	2	0.3%
2	1	0.2%
3	5	0.8%
4	4	0.7%
5	6	1.0%
6	18	3.0%
7	40	6.8%
8	103	17.4%
9	374	63.2%
10	4	0.7%
Unknown	16	2.7%
Newborn admission department		
Newborn nursery	303	51.2%
Unknown	153	25.8%
Neonatal intensive care unit	127	21.5%
Not assigned	4	0.7%
Labor & delivery	3	0.5%
Emergency department	2	0.3%
Respiratory distress diagnosis	48	8.1%
Newborn readmissions at 6 weeks		
0	561	94.8%
1	24	4.1%
≥2	7	1.2%

**Table 3:** Chief complaint categories.

	Number of ED visits	% of ED visits
Chief complaint category		
Obstetric	282	34.64%
Gastrointestinal	153	18.80%
Immunologic/infectious	108	13.27%
Neurologic	103	12.65%
Genitourinary	68	8.35%
Respiratory	60	7.37%
Cardiovascular	52	6.39%
Injury/trauma	48	5.90%
Musculoskeletal	48	5.90%
Psychiatric	40	4.91%
Constitutional	32	3.93%
Other	27	3.32%
Dermatologic	25	3.07%
Multiple complaint categories	202	24.82%
Obstetric chief complaints*		
Lower abdominal pain	97	34.4%
Vaginal bleeding	88	31.2%
Incision problem	43	15.2%
Perineal laceration	35	12.4%
Breast complaint	35	12.4%
Pelvic pain	27	9.6%
Pre-eclampsia symptoms	20	7.1%
Other	12	4.3%
Delivery	7	2.5%
Ectopic pregnancy	5	1.8%
Contractions	2	0.7%
Hyperemesis	2	0.7%
Confirmation of pregnancy	1	0.4%
Rupture of membranes	0	0.0%
Fall	0	0.0%
Decreased fetal movement	0	0.0%
Multiple obstetric complaints	58	20.6%
Pre-eclampsia symptom complaints**		
Elevated blood pressure	15	75.0%
Chest pain or shortness of breath	12	60.0%
Headache	11	55.0%
Swelling	8	40.0%
Right upper quadrant pain	4	20.0%
Vision changes	2	10.0%
Seizures	0	0.0%

* Percentage given is relative to patients with obstetric chief complaints.

** Percentage given is relative to patients with chief complaint of preeclampsia symptoms.

**Table 4:** Diagnosis categories.

	Number of ED visits	% of ED visits
Diagnosis category		
Obstetric	259	31.82%
Immunologic/infectious	229	28.13%
Gastrointestinal	126	15.48%
Genitourinary	94	11.55%
Neurologic	88	10.81%
Musculoskeletal	48	5.90%
Psychiatric	48	5.90%
Injury/trauma	45	5.53%
Cardiovascular	41	5.04%
Respiratory	35	4.30%
Other	34	4.18%
Dermatologic	27	3.32%
Constitutional	24	2.95%
Multiple diagnosis categories	263	32.31%
Specific diagnosis		
Urinary tract infection	34	4.2%
Endometritis	25	3.1%
Mastitis	25	3.1%
Pyelonephritis	20	2.5%
Headache	19	2.3%
Cholelithiasis	14	1.7%
Constipation	14	1.7%
Abdominal pain	13	1.6%
Bacterial vaginosis	12	1.5%
Postpartum depression	12	1.5%
Viral upper respiratory infection	11	1.4%
Incision infection	9	1.1%
Migraine	9	1.1%
Post-operative abdominal pain	9	1.1%
Retained products of conception	9	1.1%
Spontaneous abortion	9	1.1%

## Data Availability

The datasets generated and/or analyzed during the current study are not publicly available due to patient privacy but are available from the corresponding author on reasonable request.

## Abbreviations

ED: emergency department
UIHC: University of Iowa Hospitals and Clinics
RUCA: rural-urban commuting area
OB: obstetric
REDCap: Research Electronic Data Capture
C-section: cesarean section
UTI: urinary tract infection
